# A Parallel Comparison Method of Early Gastric Cancer: The Light Transmission-Assisted Pathological Examination of Specimens of Endoscopic Submucosal Dissection

**DOI:** 10.3389/fonc.2021.705418

**Published:** 2021-08-03

**Authors:** Guangyong Chen, Rui Xu, Bing Yue, Mei Jia, Peng Li, Ming Ji, Shutian Zhang

**Affiliations:** ^1^Department of Pathology, Beijing Friendship Hospital, Capital Medical University, Beijing, China; ^2^Department of Gastroenterology, National Clinical Research Center for Digestive Disease, Beijing Digestive Disease Center, Beijing Key Laboratory for Precancerous Lesion of Digestive Disease, Beijing Friendship Hospital, Capital Medical University, Beijing, China

**Keywords:** early gastric cancer, endoscope, pathology, endoscopic submucosal dissection (ESD), light transmission

## Abstract

**Objective:**

It is always challenging to diagnose and characterize early gastric cancer surrounded by non-cancerous mucosa, including the malignant diagnosis and extent and depth of the lesions. Therefore, we developed a light transmission-assisted pathological examination to diagnose and characterize early gastric cancer. Here, we performed a parallel comparison between the light transmission-assisted pathological examination under endoscopy and the histological examination for the diagnosis of early gastric cancer.

**Methods:**

First, the endoscopic submucosal dissection (ESD) specimen was first placed on the surface of the light-emitting diode lamp to observe the mucosal surface structure and blood vessels. Second, the sliced and embedded tissue strips were cut into 3-µm sections for hematoxylin and eosin staining. Third, the histopathology of each section was projected onto a macroscopic image. Finally, the macroscopic and microscopic changes in the ESD specimens observed under endoscopy were compared. Seventy cases of early gastric adenocarcinoma were diagnosed and characterized using this new method.

**Results:**

Using the conventional pathological method, the demarcation line of the lesions was seen in 40 of 70 (57.1%) cases. Furthermore, no surface structure or microvascular changes were observed in any of the cases. Based on the light transmission-assisted pathological examination, 58 of 70 (82.9%) cases presented clear edges of neoplastic and non-neoplastic epithelia, with a classifiable surface structure (88.6%) and microvascular type (78.8%).

**Conclusions:**

This pilot method provided a practical bridge between endoscopic and pathological examinations. Compared to the histological examination, the light transmission-assisted pathological examination was an easier and more precise way to match the *in vivo* endoscopic observation and *in vitro* pathological examination.

## Introduction

Endoscopic examination of the gastric mucosal surface structure and vascular changes is usually used to diagnose early gastric cancer (EGC). Endoscopic submucosal dissection (ESD) (1–4) is a minimally invasive procedure that achieves curative effects for early gastrointestinal cancer (5–7). The evaluation of ESD results depends on the postoperative standardized pathological examination (8). However, the lesions of EGC are often small, and changes in the mucosal surface are sometimes very slight and always confounded by the non-neoplastic changes of gastric mucosa around EGC (9–11). Moreover, the ESD specimens shrink after formalin fixation, and the color becomes darker on the mucosal surface, which makes it difficult to clearly define the lesions. During routine macroscopic examination, an unidentified area of cancer and the difficulty in determining the status of the mucosal resection margin result in an inaccurate pathological diagnosis of ESD specimens (12).

Although several different types of endoscopy are currently used to observe and study gastric mucosal lesions (13), an accurate pathological diagnosis is still required as the gold standard in verifying the specificity and accuracy of endoscopy. Because the image fields acquired by various types of endoscopes *in vivo* are small, it is difficult to make the preoperative endoscopy findings and postoperative mucosal lesions on ESD specimens have accurate “point-to-point” correspondence, which leads to a rough process and inaccurate conclusion during the comparison between the pathological diagnosis *in vitro* and gastroscopic diagnosis *in vivo* (14). Therefore, endoscopists also hope that pathologists will have better means to improve the comparison between endoscopy and pathology.

To overcome these limitations, we developed a new method based on light transmission beneath formalin-fixed ESD specimens to assist pathologists in distinguishing subtle mucosal changes in ESD specimens. We used this new method as a supplementary procedure to the routine pathological examination to make a more accurate pathological evaluation of ESD specimens. Here, we performed a parallel comparison between the light transmission-assisted pathological examination under endoscopy and the histological examination for the diagnosis of early gastric cancer.

## Materials and Methods

### Study Design and Population

In this parallel comparison study, all 70 cases were collected from the Department of Pathology, Beijing Friendship Hospital, Capital Medical University from July 2019 to October 2019. This study was approved by the institutional review board of Beijing Friendship Hospital, Capital Medical University (No. 2019-P2-133-01). The inclusion criteria were as follows: 1) intramucosal carcinoma, undifferentiated type without ulcerative findings (UL0) and ≤20 mm in size; 2) intramucosal carcinoma, differentiated types with UL0, regardless of lesion size; 3) intramucosal carcinoma, differentiated types with ulcerative findings (UL1) and ≤30 mm in size; 4) intramucosal carcinoma, differentiated types with UL0, depth of invasion of SM1, and ≤30 mm in size; and 5) high-grade dysplasia and >20 mm in size (15, 16). The exclusion criteria were as follows: 1) contraindication to gastroscopy; 2) operative contraindications to either coagulopathy or cardiopulmonary resuscitation; 3) women during pregnancy or the lactation period, or women who may not take effective contraceptive measures; 4) patients suffering from other system malignancies at the same time; 5) those unwilling to participate in this research; and 6) ESD specimens with poor fixation.

All specimens were fixed in a 4% neutral formaldehyde solution for 12 h, sectioned, and photographed with a color digital camera (Nikon D800; Nikon Corporation, Tokyo, Japan). The endoscopic examination of the lesion was performed using an Olympus GIF-290 series endoscope. White light imaging, narrow-band imaging (NBI), and near-focus magnification were used. Seventy cases of early gastric adenocarcinoma were diagnosed and characterized using our new method of light transmission-assisted pathological examination under endoscopy, all of which corresponded to the histological diagnosis.

### Endoscopic and Pathological Mutual Comparison Process

#### Step 1: Tissue Observation and Photography

**Figure 1:**
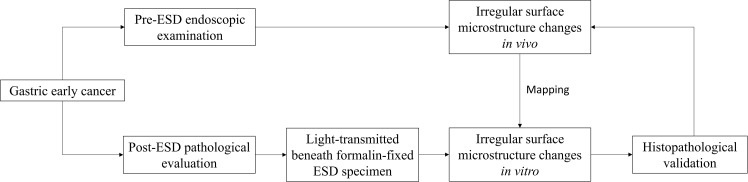
Scheme of the match comparison between the endoscopic and histopathological findings.

A light-emitting diode (LED) light was used as a light source to transmit and demonstrate the formalin-fixed ESD specimens. The parameters of the LED lamp were 3,000–6,500 K standard color temperature, 96 beads, and 15–24 W output power with adjustable brightness and color temperature. The ESD specimen was placed on the surface of the LED lamp to demonstrate the mucosal surface structure and blood vessels (3, 4). We adjusted the brightness and color temperature respectively to observe the specimen clearly. Photographs were taken separately before and after the samples were cut (**Figure 1**). The routine equipment for pathological examination of ESD specimens and the standardized procedure were used in all processes of the examination (11). After slicing, the tissue strips were embedded in paraffin and cut into 3-µm sections for hematoxylin and eosin staining according to standardized procedures (17, 18).

#### Step 2: Topographic Mapping of the Endoscopic Submucosal Dissection Specimens

Each section was numbered to map and reconstruct the ESD specimens. Next, the histopathology of each section was projected onto a macroscopic image.

#### Step 3: Comparison of the Endoscopic and Histopathological Findings

Pathologists, together with endoscopists, compared the macroscopic and microscopic changes in ESD specimens under endoscopy. Suspicious regions or areas with interesting features seen on endoscopy were linked with their histology and microarchitecture of the gastric mucosa under endoscopy *in vivo* and light transmission-assisted pathological examination *in vitro* (19, 20).

## Results

In total, 70 specimens were examined using the light transmission-assisted pathological examination. The clinicopathological characteristics of the patients are shown in **Table 1**. The boundary of the lesion, surface structure, and microvasculature were evaluated for each specimen (**Table 2**). Using routine pathological methods, clear demarcation was observed in 40 of 70 specimens (57.1%). Furthermore, it was impossible to evaluate the microarchitecture using the routine pathological method.

**Table 1:** Clinicopathologic characteristics of patients (N = 70).

Parameter	
Age, years (mean)	63 (33–79)
Sex (male:female)	42:28
Gastroscopy	
Macroscopic type	
0–IIa, IIa + I	23 (32.9%)
0–IIc, IIa + IIc, IIb + IIc	47 (67.1%)
Tumor location	
U, M	22 (31.4%)
L	48 (68.6%)
Pathology	
Size, median (range)	1.5 (0.3–5.2)
Histological classification (advantage type)	
Tub1, Pap	65 (92.9%)
Tub2	1 (1.4%)
Por, Sig	4 (5.7%)
Depth of tumor invasion	
M	53 (75.7%)
MP	9 (12.9%)
SM Vascular invasion	8 (11.4%)
(+)	15 (21.4%)
(−)	55 (78.6%)
Ulceration	
(+)	6 (8.6%)
(−)	64 (91.4%)
Number of lesion	
1	64 (91.4%)
≥1	6 (8.6%)

U, M, upper middle; L, lower; M, mucosa; SM, submucosa; MP, muscularis propria; Tub, tubular adenocarcinoma; Por, Sig, poorly differentiated adenocarcinoma and signet-ring cell carcinoma.

**Table 2:** Differences between the conventional and light transmission-assisted pathological examinations.

	Conventional pathological examination (N = 70)	Light transmission-assisted pathological examination (N = 70)
Demarcation	40 (57.1%)	58 (82.9%)
Surface structure	0 (0%)	62 (88.6%)
Microvascular change	0 (0%)	55 (78.8%)

**Figure 2:**
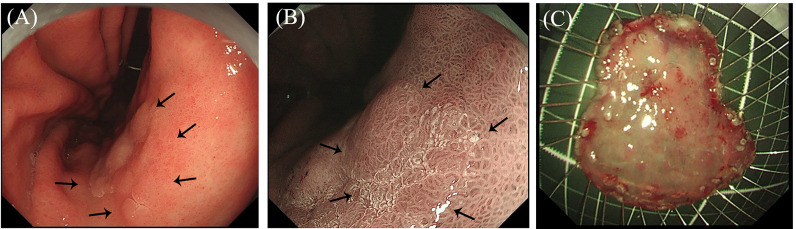
Endoscopic images of early gastric cancer. **(A)** White light imaging observations: A 25-mm flat elevated lesion is observed on the anterior wall of the gastric angle, and the center is slightly depressed (black dotted lines). **(B)** Narrow-band imaging observations: An enlarged mucosal pattern and irregular depression surface are observed (black dotted lines). **(C)** The endoscopic submucosal dissection resected specimen is fixed with a fine needle on a foam plate.

**Figure 3:**
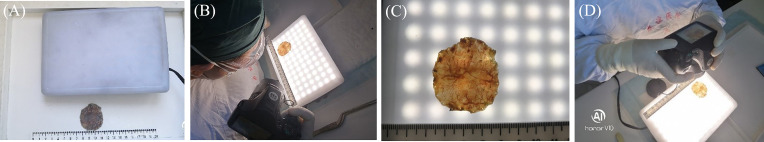
Macroscopic observation of the endoscopic submucosal dissection (ESD) specimen by light transmission-assisted pathological examination. **(A)** The light-emitting diode (LED) lamp and formalin-fixed ESD specimen. **(B)** The ESD specimen is placed on the surface of the LED lamp. **(C, D)** The LED light is transmitted through the ESD specimen, and the surface structure and vascular changes are present and photographed.

**Figure 4:**
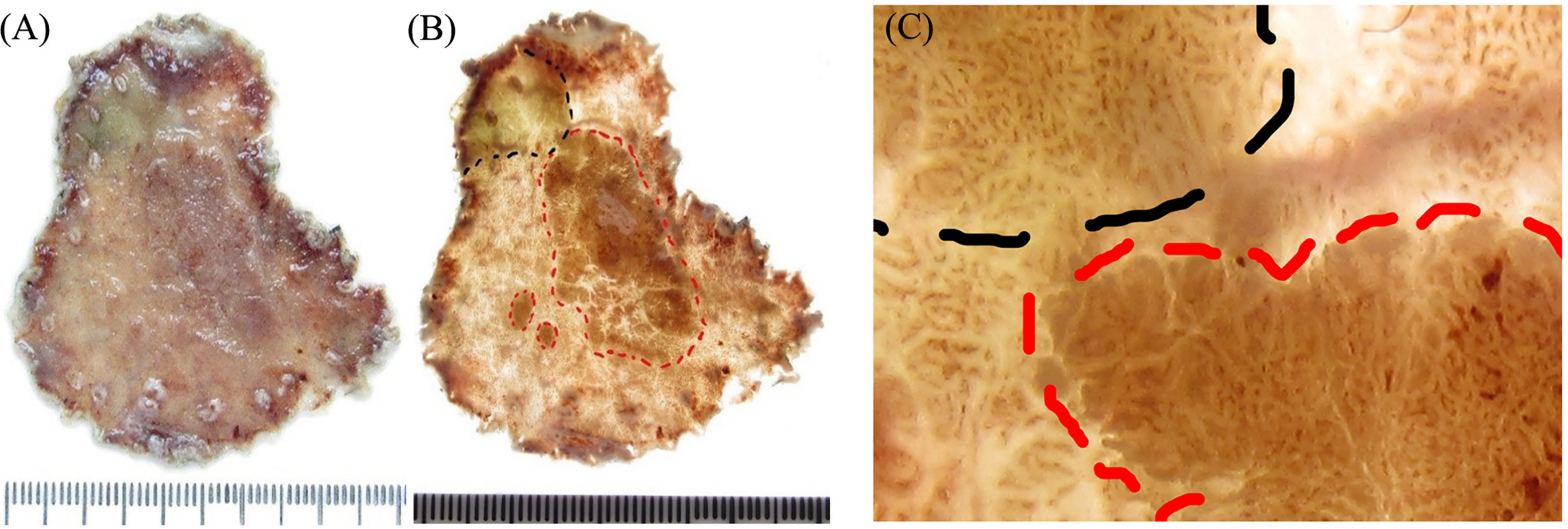
Comparison of the macroscopic specimen images between the conventional and light transmission-assisted pathological examinations. **(A)** The conventional pathological examination of the endoscopic submucosal dissection (ESD) specimen reveals a single superficial elevation of the mucosal surface with a slight depression in the center. **(B)** The light transmission-assisted pathological examination shows three distinct regions in the same ESD specimen according to the color and texture of the mucosal surface under the light-emitting diode (LED) light. **(C)** Close examination under the LED light reveals the dense mucosal microstructure and irregular microvessels of the lesion and distinguishes them from surrounding mucosa.

**Figure 5:**
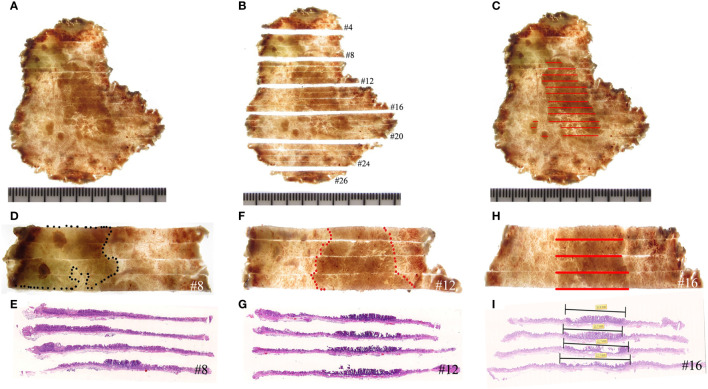
Histological mapping and corresponding hematoxylin and eosin-stained sections. **(A–C)** The photographic image is taken after cutting and sectioning the specimen. Each section is numbered to make mapping and reconstruction possible. Finally, the histopathology of each section is projected onto the macroscopic image, and topographic mapping on the endoscopic submucosal dissection specimens demonstrates three discrete areas of early cancer. **(D, E)** The specimen with histological mapping shows that the region within the black dotted line is intrinsic acidic mucosa with hyperplastic and prolonged foveola, surrounded by atrophic gastric mucosa with incomplete intestinal metaplasia, which is consistent with the gastroscopic findings. **(F–I)** Superficial elevation of the lesion is proven to be papillary adenocarcinoma by histology.

**Figure 6:**
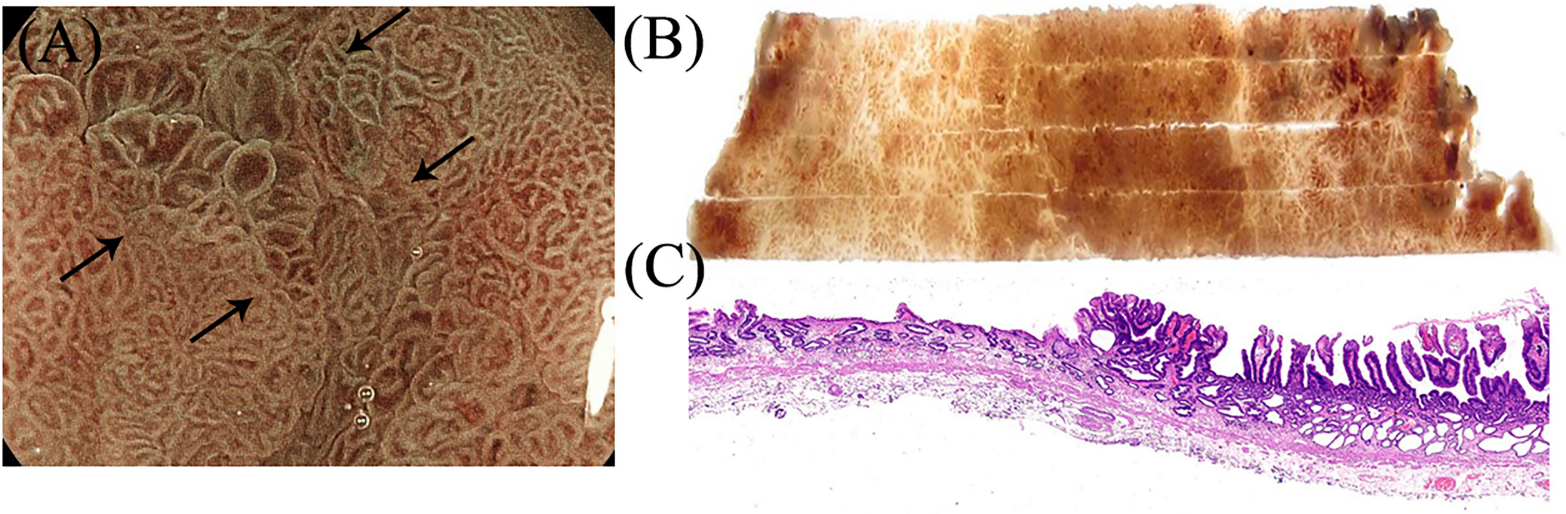
Suspicious regions or areas with interesting features seen on endoscopy were correlated with the histology. **(A–C)** Gastroscopic findings of the villous structure and irregular microvascular structure were proven to be papillary adenocarcinoma in histology.

Our light transmission-assisted pathological examination revealed clear edges of neoplastic and non-neoplastic epithelia in 58 of 70 (82.9%) specimens, and the classifiable surface structure and microvascular type were determined in 62 of 70 (88.6%) and 55 of 70 (78.8%) specimens, respectively. To better illustrate this pilot method, a case of early gastric adenocarcinoma with representative images is shown in **Figures 2**–**6**.

White light endoscopy images were collected from a 69-year-old woman with a 25 × 20-mm superficial, elevated epithelial tumor on the anterior wall of the gastric angle. Under endoscopy, the tumor was mostly light-colored, had a slightly reddened area in the central depression, and had a partially ill-defined border of the posterior wall of the gastric antrum (**Figure 2A**). The magnifying and NBI gastroscopy examination revealed irregular surface structures and vessels compared to the surrounding mucosa (**Figure 2B**). Accordingly, the lesion was diagnosed as EGC by an endoscopist and met the indication for endoscopic resection, followed by ESD. The resected specimens were fixed with a fine needle on a foam plate and soaked in a 4% neutral formaldehyde solution to be sent to the pathology department (**Figure 2C**).

The conventional pathological examination of the ESD specimen initially revealed a single superficial elevation of the mucosal surface with a slight depression in the center [Type 0–IIa + IIc, according to the Paris classification (21–23)] (**Figure 4A**). Interestingly, the method of light transmission-assisted examination was performed and showed three distinct regions that were further recognized in the same ESD specimen according to the color and texture of the mucosal surface under the LED light (**Figures 3A–D**). Specifically, one region is indicated by the black dotted lines. Another region with a brown background is indicated by the red dotted lines. The remaining region presented a light-colored brightness (**Figure 4B**). Through close examination, the region with a brown background within the red dotted lines showed papillary and tubular patterns formed by white zones, similar to what endoscopy revealed. The density of the blood vessels increased more than that in the surrounding area, in which clearly dilated vascular structures were observed (**Figure 4C**).

Consistent with the gastroscopic findings, histological mapping indicated that the region within the black dotted line was intrinsically acidic mucosa with hyperplastic and prolonged foveola surrounded by atrophic gastric mucosa with incomplete intestinal metaplasia in the specimen (**Figures 5A–E**). Gastroscopic findings of the villous structure and irregular microvascular structure were histologically diagnosed as papillary adenocarcinoma (**Figures 5F–I** and **Figure 6**).

All 70 cases were consulted for suspicious regions or areas with interesting features based on the nature, extent, and depth of the lesions under endoscopy by both pathologists and endoscopists. Among all 70 cases, 20 cases of papillary adenocarcinoma initially diagnosed by an endoscopist were eventually diagnosed with reactive gastropathy with foveolar hyperplasia. Five cases of undifferentiated adenocarcinoma were eventually diagnosed as only gastric mucosal erosion. Eight cases of abnormal gastric mucosal vessels were confirmed to have active chronic inflammation caused by *Helicobacter pylori* infection. Ten cases of EGC with indistinct edges were diagnosed as a characteristic manifestation of EGC after *H. pylori* eradication, which presented as low atypia of gastric adenocarcinomas mixed with surrounding gastric incomplete intestinal metaplasia. The other twenty-seven cases were diagnosed consistently between endoscopy and the final histopathological examination.

## Discussion

Gastric cancer sampling protocol of Japan is a useful guideline for pathologists to standardize the pathological diagnosis of ESD specimens. Following this protocol and advancements in endoscopy for decades, the ESD procedure is useful for both pathological evaluation and as a therapeutic strategy for early gastrointestinal cancer (11, 24–26). However, in practice, the EGC lesions in the ESD specimen, compared with the advanced gastric cancer specimens, were usually too small to be seen. Additionally, the mucosa around the EGC was often accompanied by atrophy, intestinal metaplasia, and hyperplasia. All of these features prevented endoscopists from judging the nature and extent of lesions and hindered the pathologists from processing the ESD specimens properly (9, 27, 28). We developed a new light transmission method, which is a better way to evaluate boundary, mucosal structure, and microvascular changes in lesions than the histological examination. This method was proven to make the ESD sampling process more targeted, avoid blind sampling, and improve the accuracy of pathological diagnosis based on Japanese guidelines (11).

Non-cancerous mucosal changes around EGC always pose a challenge for endoscopists. It is difficult to diagnose malignancy, and it is difficult to characterize the extent and depth of the lesions. Furthermore, the images acquired by various types of endoscopes *in vivo* are not easily labeled because of peristalsis and respiration. Moreover, macroscopic examination of ESD specimens using the conventional method only allows for gross classification of the lesions according to the Paris classification in most cases (21–23). However, some lesions were indistinct and poorly demarcated, and the surface structure and vessels of the mucosa could not be visualized. Thus, it is difficult to achieve an accurate point-to-point match between the areas of interest of the endoscopes *in vivo* and the corresponding mucosal lesions on ESD specimens *in vitro*. All these concerns made the endoscope–pathology match rough and inaccurate. Chemical agents, such as indigo carmine, have been tested to spray fresh ESD specimens for early colon cancer to show the mucosal surface structure changes. However, these chemical agents cannot be used to detect blood vessels (29–31). Yao et al. examined the detailed relationship between endoscopic and histological findings by investigating the length of the intervening section and the depth of the glands (3). Fujita et al. described a systematic process, the KOTO method, which allows detailed adjustments of endoscopic findings to match histopathological findings (32). The present study demonstrated that our light transmission-assisted pathological examination helped endoscopists and pathologists to easily and accurately understand the nature, depth, and extent of the lesions. This new method achieved accurate point-to-point matching and eliminated time-consuming methods needed to show the lesion. Thus, this light transmission-assisted examination provided a practical bridge between the endoscopic and pathological examinations, and compared to the histopathological examination, it serves as an easier and time-saving method to match *in vivo* endoscopic and *in vitro* pathological findings.

As a pilot, this method still needs to be improved. We noticed that the excessive thickness of the lesions, bleeding foci in some areas of the mucosa, or inflammatory exudation and fibrin covering the surface of the mucosa hindered the light from passing through the ESD specimens. To address these concerns, switching different color filters and the intensity of light transmission may be an option to help show the mucosal surface structure and blood vessels more clearly. In addition, long-term observation of the specimen under strong brightness can irritate the eye, so it is necessary to develop appropriate equipment to reduce damage to the vision of the examiner and ensure the quality of the acquired image. In the future, we anticipate that this novel light transmission-assisted pathological method based on demarcation lines, surface structure, and microvascular architecture will be applied to many other pathology specialties to improve clinical judgements of endoscopists.

## Conclusions

In conclusion, the light transmission-assisted pathology examination of ESD specimens was proven to be a useful attempt to improve endoscopy–pathology matched observations. This new method is also a bridge to improve the comparison between pathological and gastroscopic diagnoses.

## Data Availability Statement

The original contributions presented in the study are included in the article/supplementary material. Further inquiries can be directed to the corresponding authors.

## Author Contributions

GC and SZ: Conceptualization, visualization, supervision, funding acquisition, and writing–review and editing. RX and BY: Study design, validation, and visualization. MeJ: Data extraction, methodology, statistical analysis, and writing–review and editing. PL and MiJ: Methodology, validation, and visualization. All authors contributed to the article and approved the submitted version.

## Funding

This work was supported by the Beijing Municipal Science & Technology Commission (No. Z181100001718223) and the Digestive Medical Coordinated Development Center of Beijing Hospitals Authority (No. XXX0102).

## Conflict of Interest

The authors declare that the research was conducted in the absence of any commercial or financial relationships that could be construed as a potential conflict of interest.

## Publisher’s Note

All claims expressed in this article are solely those of the authors and do not necessarily represent those of their affiliated organizations, or those of the publisher, the editors and the reviewers. Any product that may be evaluated in this article, or claim that may be made by its manufacturer, is not guaranteed or endorsed by the publisher.
